# Prediction of Cranial Radiotherapy Treatment in Pediatric Acute Lymphoblastic Leukemia Patients Using Machine Learning: A Case Study at MAHAK Hospital

**DOI:** 10.31557/APJCP.2020.21.11.3211

**Published:** 2020-11

**Authors:** Amirarash Kashef, Toktam Khatibi, Azim Mehrvar

**Affiliations:** 1 *School of Industrial and Systems Engineering, Tarbiat Modares University (TMU), Tehran, Iran. *; 2 *Mahak Hematology Oncology Research Center (Mahak-HORC), Mahak Hospital, Tehran, Iran. *; 3 *AJA Cancer Epidemiology Research and Treatment Center (AJA-CERTC), AJA University of Medical Sciences, Tehran, Iran. *

**Keywords:** Acute Lymphoblastic Leukemia (ALL), childhood blood cancer, cranial radiotherapy, Prediction, MAHAK

## Abstract

**Background:**

Acute Lymphoblastic Leukemia (ALL) is the most common blood disease in children and is responsible for the most deaths amongst children. Due to major improvements in the treatment protocols in the 50-years period, the survivability of this disease has witnessed dramatic rise until this date which is about 90 percent. There are many investigations tending to indicate the efficiency of cranial radiotherapy found out that without that, outcome of the patients did not change and even it improved at some cases.

**Methods:**

the main aim of this study is predicting cranial radiotherapy treatment in pediatric acute lymphoblastic leukemia patients using machine learning. Scope of this paper is intertwined with predicting the necessity of one of the treatment modalities that has been used for many years for this group of patients named Cranial Radiotherapy (CRT). For this purpose, a case study is considered at Mahak charity hospital. In this paper, our focus is on ALL patients aged 0 to 17 treated at Mahak hospital, one of the best centers for treatment of childhood malignancies in Iran. Dataset analyzed in this study is gathered by the research team from patient’s paper-based files. Our dataset consists of 241 observations on patients with 31 attributes after the data cleaning process. Our designed machine learning model for predicting cranial radiotherapy treatment in pediatric acute lymphoblastic leukemia patients is a stacked ensemble classifier of independently strong models with a meta-learner to tune the weights and parameters of the base classifiers.

**Results:**

The stacked ensemble classifier show highly reasonable performance with AUC of 87.52%. Moreover, the attributes are ranked based on their predictive power and the most important variable for CRT necessity prediction is the disease relapse.

**Conclusion:**

In order to conclude, derived from previous studies regarding CRT it is not only cost-effective but also more healthy to eradicate the use of CRT for the treatment of childhood ALL. Furthermore, it is valuable to increase the clinical databases by creating more synthetic health databases not only for research purposes but also for physicians to keep track of their patient’s status.

## Introduction

The term “leukemia” covers a wide spectrum of blood disorders. Leukemia is classified into acute leukemia that advances quickly and chronic leukemia that progresses slowly and has got several obscure complications (Mohapatra et al., 2010). In chronic leukemia, young blood cells are present, but only the mature ones produce functional cells. Whereas, acute leukemia occurs when white blood cells are produced out of control massively that the process causes unformed, partially developed cells to be released into the bloodstream ((Rohayanti et al., 2012; Rawat et al., 2015). Acute leukemia is classified into two major classes based on a French–American–British (FAB) model, which is the most well-known classification model of leukemia: Acute Myeloid leukemia (AML) and Acute Lymphoblastic Leukemia (ALL) ((Laosai and Chamnongthai, 2014; Tran et al., 2016; Alsalem et al., 2018). Then ALL is subdivided into T-cell lymphoblastic leukemia (T-ALL) and B-cell lymphoblastic leukemia (B-ALL).

Cancer is the second most common cause of death in pediatrics and leukemia is the leading cause of death in pediatrics (Siegel et al., 2019). Due to vital improvements in supportive care treatment outcome of pediatric ALL have improved significantly over the past decades. Retrospective studies show a dramatic elevation in overall survival and nowadays ten-year survival rate is almost 90% in improved countries for pediatrics (Kersey, 1997; Wyatt and Bram, 2019); Wyatt and Bram, 2019). 

Chemotherapy was first utilized in 1948 for pediatric ALL treatment and since then several advances in this area was witnessed and these days, intensive chemotherapy and chemotherapeutics regimens are largely in action. Different pediatrics treatment centers though make use of slightly different regimens but treatment backbone and general approach are the same (Kersey, 1997; Wyatt and Bram, 2019). 

Standard treatment of acute lymphoblastic leukemia starts with four-week induction chemotherapy regimen. In order to prevent relapse, several intensive phases of chemotherapy over the course of several months, with a focus on Central Nervous System (CNS) relapse prophylaxis. Next step for the treatment is called intensification which consists of combination of chemotherapy drugs with higher doses eradicating as many blasts as possible. Final phase of chemotherapy treatment is termed maintenance, which consists of daily and weekly oral chemotherapy, monthly intravenous and oral chemotherapy, and periodic intrathecal chemotherapy. Duration of the therapy is roughly about two years for females and three years for males (Cooper and Brown, 2015; Wyatt and Bram, 2019).

Cranial irradiation was synthesized into ALL treatment after the risk for CNS relapse was recognized in the early 1980s (Cooper and Brown, 2015). Although important advances in treatment outcome was grounded due to combination of intensive chemotherapy drugs, late complications associated with cranial radiotherapy (CRT) are well-known for the world (Wyatt and Bram, 2019). Many studies have reported the negative effects of cranial irradiation (Schrappe et al., 2000; Pui et al., 2009; Vora et al., 2016), secondary malignant neoplasms and cognitive deficits and one more that we saw ourselves during data gathering was a child experienced double vision because of two courses of cranial irradiation (Wyatt and Bram, 2019). 

Secondary Neoplasms (SNs) represent serious late complications after successful treatment of malignant diseases. Löning et al., (2000) studied on 5,006 children patients with ALL divided into two groups, first group is patients who did not underwent CRT and second group is patients who was irradiated. Surprisingly, risk of SNs for those who have been irradiated was 3.5% (95% CI: 1.5%-5.5%) and significantly lower for non-irradiated patients: 1.2% (95% CI: 0.2%-2.3%). 

Pui et al., (2009) evaluated 498 ALL patients and concluded that with risk-adjusted chemotherapy, prophylactic cranial radiotherapy can be safely omitted from the treatment of childhood ALL because of potential effects after radiotherapy and better conditions regarding Complete Remission (CR) when CRT was not conducted.

To the best of our knowledge, previous studies are convincing pediatricians not to use CRT; however, many centers are still using this treatment modality in order to reduce pain or prevent the CNS relapse.

The main purpose of this study is to predict cranial radiotherapy for ALL pediatric and adolescent patients aged 3 months to 17 years enrolled and treated at Mahak hospital during 2012 to 2018, meaning that based on a patient’s demographic and clinical information we tend to predict whether it is beneficial to perform radiotherapy on a patient or not considering the risks available after irradiation. Data for this study were collected individually from paper-based records of 241 patients. Trajectory towards our purpose is paved by machine learning algorithms. Our tool in solving this problem is the powerful Rstudio and particularly we have made use of H_2_O package for the data analysis.

The paper structure is as follows: related works are discussed in the next section, after that materials of the research are being introduced and explained and prediction algorithms for our work is then explained in details. Results of the study are the next section and finally, discussion and conclusion is the ending section of this paper.

*Related works*

This area of research meaning radiotherapy prediction, particularly in ALL is completely intact making this study the world’s first pediatric ALL radiotherapy prediction. Considering this fact, in this section we will discuss radiotherapy prediction for other types of cancer. Generally speaking, studies funded until this date in this area, all aimed at predicting a complication or an event after radiotherapy treatment. Most of the studies aimed at predicting the normal tissues complications probability based on the treatment which is radiotherapy. A review article have done the grouping for these researches and formed five groups namely, Normal Tissues Complications Probability (NTCP) (Gulliford et al., 2004; Caglar et al., 2008; Spencer et al., 2009; Koiwai et al., 2010; Xu et al., 2012), Tumor Control Probability (TCP)(NAqA et al., 2010), Breathing movements (Ren et al., 2007), Survival (Gao et al., 2012), and radiotherapy effectiveness (Rockne et al., 2010). We will discuss some of the most important ones in the following paragraphs.

Radiotherapy is a primary treatment for some cancers, for instance, patients with prostate cancer would be irradiated but this radiation can cause problems such as bladder and rectum complication and (Gulliford et al., 2004) aimed at predicting these unpleasant complications with Artificial Neural Networks (ANN) using a group of 126 patients exploring good results but limited because of low number of cases. Koiwai et al. defined Total Dysphagia Risk Score (TDRS), calculated by the sum of several risk factors values. They considered a set of 47 patients with same cancer and evaluated the capacity of prediction of TDRS with Receiver Operating Characteristic (ROC) and Area Under Curve (AUC) analysis. Their analysis proved that TDRS is a valid measure for the prediction of swallowing function complications(Koiwai et al., 2010). Same objective was considered by other authors using 96 patients with head and neck cancer diagnosis. Logistic Regression (LR) was used to evaluate the relationship between dose-volume factors and swallowing dysfunctions resulting in the possibility of toxicity reduction and long term swallowing complications (Caglar et al., 2008; Anacleto and Dias, 2016).

Prediction of TCP was focused in (NAqA et al., 2010) using a database including 56 patients with lung cancer. Biological and clinical data is becoming very popular and available due to new technologies. They utilized SVM and LR in order to analyze the available data resulting in SVM merit in this particular study (Anacleto and Dias, 2016).

Predicting survival rate is a very interesting research area correspondent to different cancers. Nine different data mining algorithms were compared in (Gao et al., 2012) so as to predict the survival of two dataset of patients with colorectal cancer coming from two different sources. First dataset was composed of 10,000 registries and 20 variables, second dataset was constituted of approximately 760 registries and 14 variables. Algorithms used was Back Propagation Network (BP), Radial Basis Function (RBF), General Regression Neural Network (GRNN), Adaptive-Network Based Fuzzy Inference System (ANFIS), SVM, Bayesian Networks (BN), Naive Bayes (NB), Classification And Regression Tree (CART) and the LR. The main scope of the mentioned study was to evaluate the models precision when compared with Tumor-Node-Metastasis (TNM) system and performance measure used was AUC (Anacleto and Dias, 2016).

A model that tries to predict the radiotherapy effectiveness in a pilot study was managed using a classic linear-quadratic (LQ) approach. Database for this study was consisted of 9 patients with glioblastoma. Major purpose was to define a comprehensive model for invasion of the gliomas affected by radiotherapy (Rockne et al., 2010; Anacleto and Dias, 2016).

Despite all these glamorous researches, to the best of our knowledge, no study was found considering the prediction of radiotherapy based on patient’s clinical and medical information. This gap is fulfilled in this article as we aim to predict whether radiotherapy treatment is needed for a particular patient or not based on features that we included in our data. One more important feature of this study is that we considered pediatric acute lymphoblastic leukemia patients which has been never done before, to the best of our knowledge. Moreover, the attributes are ranked based on their predictive power on requiring radiotherapy treatment for a particular patient suffering from ALL or not.

## Materials and Methods

**Figure 1:**
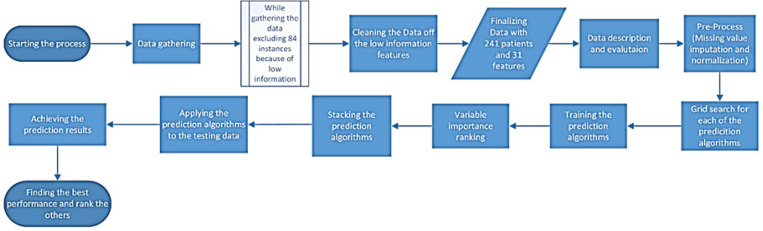
The Flowchart of the Main Steps have been Used in the Research Methodology

In this study we are making use of machine learning techniques in order to predict the requirement of CRT for pediatric acute lymphoblastic leukemia patients aged from less than 1 to 17 years old treated at Mahak hospital from 2012 to 2018. In this section we will explain the features of the dataset being used in this study and walk through the methodology we tend to use for reaching our purpose. Figure 1 shows the main steps of the research methodology used in this article. 

As shown by Figure (1), more details on the main steps of the research methodology are described in the following subsections.

*Data gathering*

Data gathering procedure took four months collecting all the data from paper-based records from two sources, clinical file and inpatient file. Basic and demographic data was collected from clinical file and more medical and detailed data from inpatient file. Clinical file is a briefed version of medical records for the patients but inpatient file is a comprehensive and day by day history of the patients where every transcription is available and every daily event is recorded. We thoroughly read all the sources for the patients and extracted the data. Also, all the attributes of the data were considered after an authorization from a group of experts in Mahak hospital. Furthermore, while gathering the data we intended to decrease the preprocessing by excluding observations with low amount of available data which finally resulted in 241 patients and 31 attributes. Next section corresponds to the different features of the data.

*Data description*

**Figure 2:**
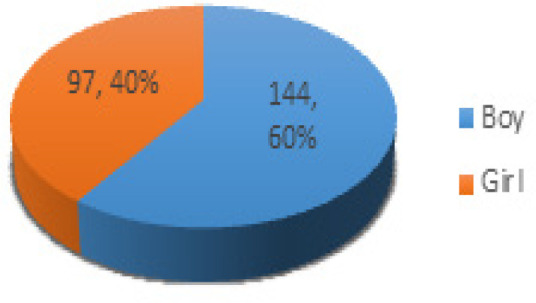
Sex Distribution of ALL Patients in the Data

Data which we gathered is composed of 241 observations and 31 features after cleaning the constant and useless variables. For instance, to our knowledge chemotherapy is a standard treatment and every patient go through chemotherapy so it is a constant variable and should be omitted. Moreover, demographic features are limited to sex and age at the time of diagnosis and the rest of the features corresponds to medical and clinical features. ALL is widely documented and witnessed amongst pediatrics and according to (RUBNITZ and PUI, 1997; Nevine M. Labib, 2005; Morton, 2010; Siegel et al., 2019; Wyatt and Bram, 2019) affecting male population more than female population and once more based on our data this fact is seen transparently as Figure 2 shows this phenomenon. It can be seen from the figure 2 that 97 patients (40%) are girls and 144 patients (60%) are boys.

**Figure 3:**
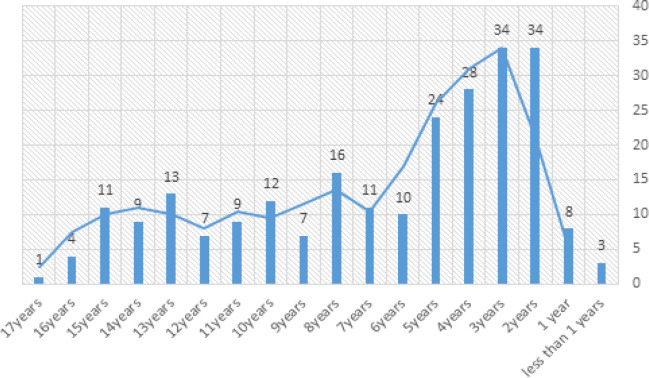
Age at the Time of Diagnosis Distribution in Our Data

History has shown us that children at the ages of 2 to 5 are at high risk of being diagnosed with ALL (Belson et al., 2007; Siegel et al., 2019), patients involved in our data range from three months old to 17 years old, figure 3 is the age distribution of patients involved in our data.

As it is clear from the figure3 most number of incidences was for children at two and three years of age with 34 cases equally. Four major and important components of blood which are White Blood Cells (WBC), Red Blood Cells (RBC), Platelets (PLT) and Hemoglobin (HG), are included in the data. Mahak experts and our team decided to record the number correspondent to each of the major blood components in the first blood trial that based on which pediatricians and oncologists first diagnosed the ALL. Numbers vary significantly, WBC from 500 to 284,000, RBC from 274,000 to 5,640,000, PLT from 5,000 to 955,000 and HG from 2.4 gr/dL to 16.2gr/dL. Common type of ALL is the B-lineage type and is worldly known as B-cell ALL and more intense and rare type is the T-cell ALL, this fact is strongly founded in our data. Figure 2 illustrates the majority of each type of ALL.

It can be seen from the Figure 2 that 216 out of 241 (89.6%) had the common ALL and 25 patients (10.4%) were suffered from T-cell ALL.

Next, is the risk group for each patient which based on Mahak hospital regulations and instructions this variable is at three stages, Standard Risk (SR), Intermediate Risk (IR) and High Risk (HR). Figure 3 shows the patient’s proportion correspondent to each risk group. 

From 241 patients in the data, 14 (5.8%) were treated with Allogeneic Stem Cell Transplant (Allo-SCT) but unfortunately six of those died after a while and eight patients survived from the disease. CRT is one of the alternatives in the treatment of pediatric ALL and based on the data 27 (11.2%) patients underwent at least one course of CRT in order to prevent CNS relapse or eradicate CNS relapse footprints. Furthermore, paying attention to chemotherapy drugs have been used in the treatment, one drug named L-asparaginase is one of the most essential drugs in the treatment protocol. In the intensification phase of chemotherapy when the correspondent physicians increase the dosage of the drugs a proportion of patients show allergy specifically to L-asparaginase. This phenomenon was recorded in the data showing 12 patients who showed an inverse reaction to the mentioned drug. Other features are a set of 17 most important treatment-related complications such as pneumonia, neutropenia, mediastinal mass for T-cell patients, fever and Graft-Versus Host Disease (GVHD) to name but a few (Pommert et al., 2019). 

*Data preprocessing and preparation*

In machine learning, data preprocessing is one of the most significant parts and researchers are bound to operate this phase properly in order to achieve reasonable results from every data. Accordingly, in this study we aimed at decreasing this process when gathering the data; however, missing values are inevitable. So in this study we have done the missing value imputation using missforest package in Rstudio (Stekhoven, 2012) because it deals with every type of data, resulting in 0.129 out of bag error.

Next, Normalization was done resulting every value in the data a number between 0 and 1. Following that with dividing the data into two parts, train and test and that was done with 60% and 40% split respectively, creating train set with 138 observations and test set of 103 observations. Furthermore, we set aside 30 instances from train set for evaluation purposes. 

*Grid search*

Cartesian grid search was implemented using H_2_O sub functions for each model before the model is trained in order to trace the best suited hyper parameters. Hyper-parameters are a primary source for model credibility enhancement achieving the most optimized result. A model hyper-parameter is a characteristic of a model that is external to the model and whose value cannot be estimated from the data. The value of the hyper-parameter has to be set before the learning process begins. That is all the reason why we implement the grid search for the prediction algorithms. Grid-search is used to find the optimal hyper-parameters of a model resulting in the most accurate predictions (H2o.ai, 2020a).

*Prediction algorithms*

In this study we are making use of a powerful library named H_2_O developed by H_2_O.ai, a visionary Silicon Valley open source software company. There are a lot of machine learning algorithms covered by H2O package and we intend to make use of three of the supervised algorithms in our study namely, Distributed Random Forest (DRF), Gradient Boosting Machine (GBM)(Candel, 2020) and, Generalized Linear Model (GLM)(Tomas et al., 2020). Version of the h2o package used is 3.28.0.3. Furthermore, AUC is the evaluation metric based for comparing the models finding the best performance. Besides, accuracy, prediction and recall on test set is reported for every trained model. Also all the models are implemented using 5-fold cross-validation.

In order to stack basic models they need to be cross-validated with the same number of folds and the keep_cross_calidation_prediction parameter set to true. In this case, we need basic algorithms that could be cross-validated (H_2_O.ai, 2020b). Among supervised algorithms covered by H2o package there are deep learning by neural networks, DRF, GLM, GBM, naïve bayes classifier, SVM and XGBoost. First of all, SVM cannot be cross-validated in H2O package and then XGBoost is not supported by windows in this version. Naïve bayes is most useful when there are more than two labels in the response column and in fact deep learning is not very practical on every dataset and the data used in this study is one of those datasets because it is not big enough to get any profound effect from deep learning method. Remaining methods for our purpose are DRF, GLM, and GBM.

*Stacked ensemble*

Ensemble machine learning methods use multiple learning algorithms to obtain better predictive performance than could be obtained from any of the constituent learning algorithms. Many of the popular modern machine learning algorithms are actually ensembles. For example, Random Forest and Gradient Boosting Machine (GBM) are both ensemble learners. Both bagging (e.g. Random Forest) and boosting (e.g. GBM) are methods for ensembling that take a collection of weak learners (e.g. decision tree) and form a single, strong learner (H_2_O.ai, 2020b).

H_2_O’s Stacked Ensemble method is a supervised ensemble machine learning algorithm that finds the optimal combination of a collection of prediction algorithms using a process called stacking. Like all supervised models in H_2_O, Stacked Ensemble supports regression, binary classification and multiclass classification (H_2_O.ai, 2020b).

Once all the prediction algorithms were learned we created a stacked ensemble model for every set of algorithms possible. Finally, we would have four stacked ensemble models with base learners of independently strong algorithms.

*Evaluation and validation measurement*

In order to evaluate the performance of the prediction algorithms, four different evaluation metrics are taken into action. Basically, AUC is used for the comparison with which we ranked the prediction algorithms and other three metrics are reported. In this section we take a quick look at the formulas of each evaluation metric namely, accuracy, precision, recall, and AUC.

Eq.1) Accuracy= (TP+TN)/(TP+TN+FP+FN)

Eq.3) Recall= TP/(TP+FN)

Eq.2) Precision= TP/(TP+FP) 

Eq.4) AUC= (TPr+TNr)/2 

## Results

Results achieved from the analysis show a strong link between clinical data and prediction of CRT treatment for children suffering from ALL. Table 1 shows the results obtained from each of the prediction algorithms after running the Cartesian grid search. Which means these are the most optimized results for each of the models. Furthermore, Table 2 represents the best suited hyper parameters based on our data achieving the results showed in Table 1.

Additionally, for the GLM we implemented a random grid search instead of Cartesian because alpha and lambda are between 0 and 1 and is very tricky to do the Cartesian mode. Finally, it can be seen that GBM independently has got the best performance for the CRT prediction. Even though GLM delivered the highest accuracy, it is showing the lowest AUC and as we set AUC the comparison factor GLM has the weakest performance with 0.8347 AUC and GBM has the best performance with 0.8659 AUC. It is worth mentioning that the validation set has got two records of the positive label and 28 of the negative one. 

Then, these three models were stacked forming four set of combination resulting in four new models which were created by stacking three independently strong models in order to achieve better prediction. For learning a stacked ensemble model we need to set some parameters and find the most suited value for each parameter in order to obtain the best results than the single base models. We found the most pragmatic values for parameters on each stacked model and fortunately succeeded to increase the AUC. Table 3 compares the results achieved from four different stacked models. Besides, these stacked ensemble models were implemented using 5 fold-cross validation as well as basic models. 

Eventually, after stacking three independently strong prediction models it can be seen that the best performance was for stacking GBM and DRF resulting in AUC of 0.8752 which is a significant increase in healthcare domain from 0.8659. Furthermore, accuracy saw a dramatic increase from 88.35% with GLM to 91.26% with stacking all three models together. Finally, by stacking GBM and DRF we could achieve the best performance and that best performance is an AUC of 0.8752 and accuracy of 90.29% with 0.309 threshold. Table 4 illustrates the major parameters for the metalearner of the stacked ensemble of the model with the best performance.

Moreover, Table 5 is the confusion matrix resulted from GBM and RF stacked ensemble prediction model.

Additionally, MSE and RMSE for the best prediction model which is a stacked ensemble model including GBM and DRF algorithms as the basic algorithms are 0.0922 and 0.3037, respectively. Other metrics including logloss, AUCPR and gini are measured for the best prediction model and their value are 0.3697, 0.5277 and 0.7504 respectively. 

Furthermore, variable importance report was not available through a stacked model so we report the variable importance from the GBM model which is very close to the best model here. Table 6 demonstrates the top 10 variables for the prediction implemented using GBM.

According to experts insights and recommendations, a child who experienced at least one time relapse during the therapy and particularly Central Nervous System (CNS) relapse are qualified to undergo at least one session of CRT and of course this fact is well identified and extracted with our data mining process finding the relapse variable the most important factor for CRT necessity prediction. Second most important variable identified from the data mining process is the cell type meaning the lineage of the ALL either it is B-cell or T-cell and obtained from our data is the fact that mostly the B-lineage ALL children, precisely two times the T-cell patients, received CRT for therapy purpose. Third most important variable for predicting the necessity of CRT is the age at the time of diagnosis, where the youngest was two years old and oldest 16 years old.

## Discussion

Datasets and availability of the suitable data is the cornerstone to every data mining research. Most of the limitations and restrictions for not achieving the best possible result is due to dearth of enough observations. Typically, clinical data is collected in the course of patient care, many times in a manual way, while the necessary research data are forgotten or left for second plan. Therefore, the clinic databases can present wide comprehensive information when taken seriously. The attributes that are available in the datasets are another important feature that has to be considered and selected wisely. Having a wider set of attributes makes possible the use of variable selection approaches, that will allow a better selection of prediction variables that, hopefully, will bring more and better insights regarding the potential relationships that exist between dependent and independent variables (Xu et al., 2012). Having enough data to feed data mining models is crucial if we want to obtain higher quality results leveraging the possibility of knowledge retrieval and generalization. The data might have missing values or noise, be imprecise, redundant or inconsistent. Accordingly, considering this fact, in this present study we ran the anomaly detection with h2o package and omitted those observations with MSE higher than 0.1 but we encountered a drop in the results, hence we decided not to omit any observation mostly because even one observation in the health domain worth more than to be accounted as anomaly.

This study is a unique of its own for the childhood ALL and was never done with this scope. We examined and found out that this area regarding the necessity of a particular treatment modality prediction is worth investigating. Another finding that we led to was that CRT in childhood ALL would not lead to death but it may be accompany the patient with side effects. In our study there were 13 patients who did not underwent CRT but eventually died, on the other hand there were 18 patients who survived from the cancer but received CRT.

Data for this research have been gathered by the research team so it is very carefully collected and evaluated but its very time consuming to collect data for every research. Clinical data such as the data for this very study can expand the knowledge and insights through different life-threatening diseases; however, many health centers and hospital do not record the data electronically in order to use in the researches and in order to overcome this limitations synthetic databases can be created so as to increase the speed of making knowledge in different areas.

Moreover, this study is very significant because there were many studies investigating the role of CRT in the treatment of childhood ALL patients (Löning et al., 2000; Schrappe et al., 2000; Pui et al., 2009; Vora et al., 2016). All unanimously reported that outcome of the acute lymphoblastic leukemia patients without CRT would enhance based on experiments. Accordingly, in this study we aimed at finding the necessity of CRT for childhood ALL patients and succeeded at prediction of CRT necessity at 90 percent accuracy approximately. Combining those articles investigated at CRT necessity found that no precise need is required with our study, it is sensible that physicians and pediatricians may pay serious attention to those patients who their disease relapses and try to experiment new treatment modality rather than CRT in order to prevent the potential complications. 

Finally, where we conducted our study there is a procedure for a patient to be gone through before starting to be treated with CRT. This particular flow starts from the correspondent physician offering a consultant from radiologist when watching a set of conditions in the patient such as CNS relapse then, the radiologist assesses the patient and if the patient’s conditions are suitable for the CRT to be started, precise date will be set for the first and the last session of the CRT. Accordingly, when we perfectly succeeded at predicting whether a specific patient requires CRT, this procedure is optimized and the time particularly is saved. It means that with this study we have made the procedure time-effective.

In conclusion, CRT is one common treatment modality that has been used for ALL patients, in particular childhood ALL. Proved in many investigations that not only CRT would not help the patient through his or her treatment, but also it may cause lifelong complications. In this technological era, machine learning is widely utilized in many domains especially healthcare for disease controlling. In this study we made use of powerful package in Rstudio named h2o which has the ability to cover most of the machine learning models along with deep learning models. Link between machine learning tool and healthcare domain is created in this actual study as we aimed at predicting the necessity of CRT for children suffering from ALL. Prediction of the necessity of CRT is highly vital in order to minimize the use of CRT and is done in this study and we succeeded very perfectly in that purpose as we managed to predict the use of CRT with 0.8752 AUC and 90 percent accuracy identifying relapse as the most important variable and besides making the CRT procedure time-effective with this prediction. Furthermore, the use of ensemble methodologies could also improve the accuracy of prediction models as we performed the stacked ensemble model and improved the model performance. Eventually, it would be beneficial if the health authorities manage to create a more synthetic database in order to increase the speed of making new knowledge out of big amount of data available these days. 

**Table 1:** Evaluation Metrics Achieved from Prediction Algorithms

Prediction algorithms	AUC (test set)	Threshold	Max Accuracy (test set)	Threshold	Max Precision (test set)	Threshold	Max Recall (test set)	AUC (validation set)
GBM	0.8659	0.1946	87.38%	0.1946	100%	0.0473	100%	0.89
GLM	0.8347	0.1368	88.35%	0.1819	100%	0.0843	100%	0.785
DRF	0.8483	0.2276	85.44%	0.072	45%	0.0272	100%	0.803

**Table 2:** Best Suited Hyper Parameters for Each of the Prediction Algorithms

Gradient Boosting Machine (GBM)	Random Forest (DRF)	Generalized Linear Model (GLM)
Learn rate = 0.01	Ntrees = 100	Alpha = 0.0186
Sample rate = 0.8	Mtries = 7	Lambda = 0.963477
Ntrees = 50	Max depth = 7	
Col sample rate = 1	Sample rate = 0.2	
Max depth = 3		

**Table 3:** Evaluation Metrics Achieved from Stacked Ensemble Models

Prediction algorithms	AUC (for test set)	Threshold	Max Accuracy (test set)	Threshold	Max Precision (test set)	Threshold	Max Recall (test set)	AUC (validation set)
GBM & GLM	0.8142	0.4058	89.32%	0.4058	80%	0.00065	100%	0.8571
GBM & DRF	0.8752	0.3095	90.29%	0.6881	100%	0.000931	100%	0.8214
GLM & DRF	0.8338	0.0397	85.44%	0.000259	45.45%	0.000001	100%	0.7857
GBM & GLM & DRF	0.8732	0.4122	91.26%	0.752138	100%	0.000766	100%	0.8214

**Table 4:** Metalearner Parameters of the Best Stacked Model (GBM and DRF)

Metalearner algorithm	Metalearner fold assignment
"gbm"	"Random"

**Figure 4:**
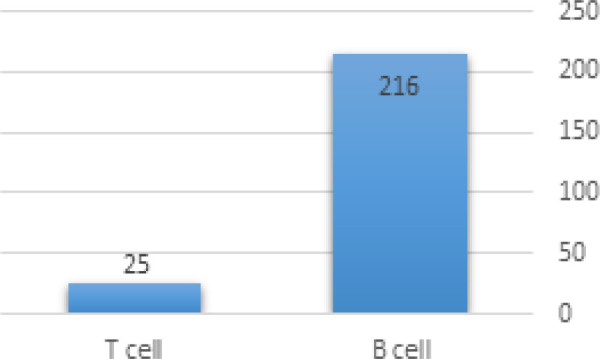
Majority of Each Type of ALL in the Data

**Table 5:** Confusion Matrix Resulted from GBM and RF Stacked Ensemble

Confusion Matrix			Error	Rate
	0	1		
0	75	14	0.1573	14/89
1	2	12	0.1428	14-Feb
Total	77	26	0.1553	16/103

**Table 6:** Top 10 Variables Derived from GBM Prediction Model

1	"Relapse"
2	"Cell type"
3	"Age at diagnosis"
4	"Platelets"
5	"Risk group"
6	"fever"
7	"Pneumonia"
8	"Hemoglobin"
9	"Immunocompromised condition"
10	"Red Blood Cells"

**Figure 5:**
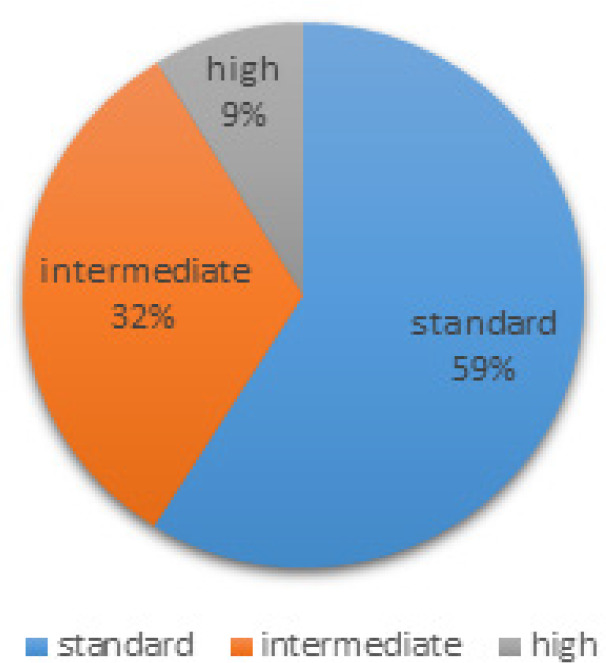
Percentage of Patients in Each Risk Group
